# Current state of research on psychotherapy for home-living vulnerable older adults with depression

**DOI:** 10.1007/s00391-020-01805-3

**Published:** 2020-11-13

**Authors:** Christina Tegeler, Ann-Kristin Beyer, Fee Hoppmann, Valentina Ludwig, Eva‑Marie Kessler

**Affiliations:** 1 Department of Psychology, MSB Medical School Berlin, Rüdesheimer Str. 50, 14197 Berlin, Germany; 2 Institute for Medical Sociology and Rehabilitation Science, Charité—Universitätsmedizin Berlin, Charitéplatz 1, 10117 Berlin, Germany

**Keywords:** State of the art review, Frailty, Homebound individuals, Psychosocial interventions, Collaborative care, State-of-the-Art-Review, Frailty, Menschen mit Pflegebedarf, Psychosoziale Interventionen, Integrierte Versorgung

## Abstract

Older home-living vulnerable adults often suffer from chronic conditions accompanied by restrictions in mobility, social participation and reduced independence. Among this rapidly growing population depression is a common and serious health problem; however, there are shortcomings in the diagnosis of depression and provision of psychotherapy. Despite growing evidence in treating depression among the group of nursing home residents or the “young-old”, there is a research gap regarding needs-oriented healthcare strategies for very old, frail or care-dependent older adults living at home. The present article provides an overview of different outpatient psychotherapeutic treatment approaches for vulnerable older adults with depression, in particular adaptations tailored to those who are homebound or in need of care. Based on the current state of research, this article derives recommendations for psychotherapy in this special setting to consider the physical and psychosocial resources of this patient group. Furthermore, healthcare strategies for embedding psychotherapy in collaborative, telehealth or home-delivered healthcare services are described and their applicability as psychosocial support for older adults during the coronavirus disease 2019 (COVID-19) pandemic is discussed. Psychotherapy is an efficacious treatment for depression in home-living vulnerable older adults. Further implementing telehealth or home delivered settings, individually tailored psychotherapeutic approaches as well as collaborative and stepped care approaches can increase utilization and medical supply of this patient group. More research and innovative programs are needed to improve access to and provision of psychotherapeutic care as well as their social inclusion.

## Abstract (de)

Ältere vulnerable Menschen leiden neben gesundheitlichen Beeinträchtigungen häufig unter Einschränkungen der Mobilität, sozialen Teilhabe und selbstständigen Lebensführung. In dieser wachsenden Bevölkerungsgruppe sind depressive Erkrankungen ein häufiges und folgenschweres Gesundheitsproblem, das jedoch häufig nicht erkannt wird. Auch Psychotherapie wird kaum vermittelt. Trotz Forschung im stationären Setting und zu „jungen Alten“ mangelt es weiterhin an bedarfsgerechten Versorgungsangeboten für sehr alte, gebrechliche und pflegebedürftige ältere Menschen, die zu Hause leben. Dieser Artikel gibt einen Überblick über verschiedene ambulante Psychotherapieansätze zur Behandlung vulnerabler älterer Menschen mit depressiven Erkrankungen, insbesondere bei Immobilität oder Pflegebedarf. Orientiert am derzeitigen Forschungsstand werden Empfehlungen für die Gestaltung von Psychotherapien abgeleitet, um die körperlichen und psychosozialen Ressourcen der Patient*innen differenziert zu berücksichtigen. Versorgungsmodelle zur Einbindung psychotherapeutischer Interventionen in kooperative, aufsuchende und telemedizinische Versorgungsmodelle werden vorgestellt und in Hinblick auf ihr Potenzial zur psychosozialen Unterstützung älterer Menschen während der COVID-19-Pandemie diskutiert. Psychotherapie ist eine effektive Behandlungsmöglichkeit von depressiven Erkrankungen bei zu Hause lebenden, vulnerablen älteren Menschen. Der Einsatz von telemedizinischen oder aufsuchenden Settings sowie die Versorgung durch kollaborative oder Stepped-Care-Ansätze kann die medizinische Inanspruchnahme und die Versorgung dieser Patient*innengruppe fördern. Weitere innovative Programme und Forschungsansätze sind notwendig, um ihre Versorgungssituation und gesellschaftliche Inklusion zu verbessern.

Depression is a common mental disorder in later life. An international meta-analysis found a pooled prevalence of 7.2% (95% confidence interval, CI 4.4–10.6%) for major depression and 17.1% (95% CI 9.7–26.1%) for depressive disorders among adults 75 years and older [29]. Within the group of homebound older adults and those receiving home healthcare, depression rates increased by a factor of two or three have been reported [12]. A strong reciprocal relationship exists between depression and chronic physical conditions. Specifically, highly prevalent physical health problems, functional limitations and chronic conditions in older age are often accompanied by depression. Conversely, depression increases the risk of morbidity, frailty and mortality. Furthermore, untreated depression also results in higher rates of healthcare service utilization and even premature institutionalization [35, 36].

The limitations of antidepressant medication (e.g. reduced response and adherence rates, drug-drug interactions and adverse side effects [37]) in older adults and evidence for a strong preference for psychotherapy [28] indicate a higher need for psychotherapeutic services for homebound and vulnerable older adults with depression. Studies and meta-analyses have shown that several psychotherapeutic treatments are effective in treating depression in older adults [18, 30]. In recent years, an increased amount of research on psychosocial and psychotherapeutic interventions to combat depression among nursing home residents has been conducted [5] including the prominent manualized programs BE-ACTIV [31] and R‑E‑M (restore-empower-mobilize; [9]); however, many of these studies excluded vulnerable older adults including homebound adults and those with multimorbidity or dementia [13]. Fewer programs have been implemented for the even harder to reach population of home-living vulnerable older adults. Consequently, empirical evidence supporting psychotherapy for this rapidly growing population has barely been conducted.

Analogous to this shortage of research, patients in this population are almost always excluded from psychotherapeutic services even in regions with high availability of psychotherapeutic resources such as Germany [15]. There are a range of barriers regarding access to psychotherapy that particularly affect home-living vulnerable older adults. Deficit-oriented perceptions of mental health in old age are still prominent within individuals and healthcare providers [21]. The lack of barrier-free and accessible healthcare institutions, shortage of psychotherapists qualified to work with older adults or difficulties with arranging home visits are examples of structural barriers that may in part reflect negative views towards psychotherapy in old age [26].

The described deficiencies in research and healthcare supply and lack of reliable evidence result in largely unmet psychotherapeutic needs of home-living older vulnerable patients. Therefore, appropriate psychotherapeutic approaches for treating depression and adequate healthcare services as well as means to overcome the mentioned barriers are necessary. The present article provides an overview of the current status of international programs, healthcare strategies and clinical recommendations relating to psychotherapy for home-living vulnerable older adults with depression. By vulnerable older adults, we refer to those encountering certain physical challenges and functional limitations, such as chronic illnesses, limited mobility, sensory impairments, frailty and cognitive decline, including the population of people in need of care and those who are homebound. While in this article we do not explicitly consider social vulnerability as indicated, for example, by factors such as low income or living in rural areas, which exacerbate poor treatment supply in this patient group. Considerations for specific adaptations of psychotherapeutic treatment and evidence-based approaches tailored to the needs of this patient group are derived and healthcare strategies embedding psychotherapy into collaborative care and telehealth are described. Given the presumably massive implications of the coronavirus disease 2019 (COVID-19) pandemic on the mental health of older adults [4], we also discuss how psychosocial support could be realized for vulnerable older adults in the current situation and beyond.

## Methods

Electronic database searches were undertaken using MEDLINE, PubMed, PsycINFO and Cochrane Library in April and May 2020 with no time restrictions. A backwards search for references to potentially relevant articles was also conducted. The search terms included words related to older adults with depression, need of care and psychotherapy (cognitive behavioral therapy, CBT, collaborative care, geriatric, geriatric depression, homebound, late-life depression, multimorbidity, need of care, old age, primary care, psychotherapy, telehealth, vulnerable) as well as COVID-19. Articles, reviews, and book chapters were considered as document types if they were written in English or German. They had to include programs, healthcare strategies or clinical recommendations for home-living vulnerable older adults with depression, outpatient psychotherapeutic treatment approaches, especially in the case of mobility limitations and intervention studies with randomized controlled trials, as far as possible.

## General considerations for psychotherapy with home-living vulnerable older adults

Home-living vulnerable older people are a distinct but very heterogeneous patient group when considering the wide range of complaints, resources and needs. Psychotherapy with very old, frail and care-dependent older adults has not been thoroughly researched; however, for treating depression in older adults in general, most standard psychotherapeutic approaches and methods appear to be useful when applied in an age-sensitive manner by informed practitioners (for professional practice guidelines see [2]). In the following, we present an informed selection of recommendations derived from the literature (e.g., [2, 22]) as well as our clinical expertise on beneficial treatment adaptations to address specific needs of chronically ill or homebound patients.

### Psychotherapeutic attitude.

It has been reported that psychotherapists perceive a pronounced need for safety, attachment and intimacy in many frail older patients, which should be addressed by allowing for higher than usual emotional closeness in the psychotherapeutic relationship [22]. Furthermore, it is necessary to pay close attention to validating patients’ individual characteristics and needs [14]. Especially with older adults who are in need of care, age bias may impede adequate psychotherapeutic treatment. According to survey studies and experimental studies using patient vignettes, psychotherapists are less inclined to treat older patients and may doubt their treatability [20, 25]. Therefore, psychotherapists need to become aware of their age biases [27] and promote their patients’ resources and sense of autonomy (see [21] for an overview on individual views on ageing in the psychotherapeutic context).

### Common themes.

Certain matters have been reported to be more likely to emerge and to be encountered in psychotherapy with older adults and might be of particular interest for homebound or vulnerable patients. Examples are loss (e.g. losses of spouses, friends, functions or independence) and life transitions (e.g. moving from one home to another, accepting care services or devices or adapting to new roles in the family), ageing and ageism as well as physical health complaints, social isolation or interpersonal conflicts. Psychotherapy can provide a trusting relationship to process those issues [3].

### Physical health and diagnostics.

Diagnosing depression in older adults with chronic conditions requires an exchange between physicians and psychotherapists to recognize and differentiate diverse medical, psychological and social etiological factors of the presenting complex symptomatology. Side effects and interactions of multiple drugs can cause symptoms akin to depression (for more information, see [7, 37]). Furthermore, diagnosticians need to be aware of age-specific expressions of depression symptoms (e.g. strong anhedonia, difficulties with concentration and memory opposed to the core symptom of sadness typical in younger patients) and consider some patients’ tendency to trivialize painful experiences. Even if correctly detected, patients suffering from depression might not be referred to psychotherapy due to symptoms being falsely perceived as an adequate response to life circumstances [14].

### Caregiver involvement.

Special consideration should also be given to the inclusion of professional and/or informal caregivers as well as relatives. Caregivers are often under considerable psychological strain and the relationship may be burdened by dependence and conflicts [46]. Psychotherapists can act as facilitators by referring support systems, imparting knowledge about depression, moderating solution focused communication or supporting problem solving behavior.

### Setting.

A psychotherapist’s practice needs to be accessible, barrier-free and adjusted to physically impaired patients’ needs (e.g. chairs suitable for patients with pain, material for sensory impaired patients, lifts instead of stairs). Alternatively, psychotherapists should consider home visits. These offer genuine impressions of the patient’s reality and the opportunity to practice new behavior directly in their living environment. Home visits require psychotherapists to be flexible and work in an unfamiliar setting as well as taking on multiple roles (guest and host) at the same time [22]. In this setting, the maintenance of professional distance can be more challenging. Home provision entails time and cost for the journey to the patients and may thus cause economical disadvantages for the psychotherapist or the patient unless adequately supported by healthcare providers [14, 22].

## Evidence-based psychotherapeutic treatment approaches

Systematic reviews and meta-analyses have provided evidence for several psychotherapeutic interventions to be effective for treating depression in older adults, with strongest evidence for cognitive and behavioral therapy (CBT), life review therapy (LRT), interpersonal therapy (IPT) and problem solving therapy (PST) (for more information see [18, 30]). Well-controlled studies on the effect of psychodynamic psychotherapy (PDT) with older people are still sparse but previous evidence indicates positive effects [42]. Table 1 provides an overview of those approaches and relates them to home-living vulnerable patients.

**Table 1:** Evidence-based psychotherapeutic treatment approaches for older adults

Approach	Description
*Cognitive behavioral therapy (CBT)*	CBT includes a wide range of therapies that focus on the detection and modification of dysfunctional (cognitive, behavioral, emotional and motivational) behavior. Typical primary goals in the depression treatment of home-living vulnerable older adults are to support the patients’ maintaining or regaining of their self-determination, to engage more often in pleasant activities and to implement helpful coping strategies for stressful situations. Low intensity versions of CBT have been developed as short, manualized interventions with flexible delivery and adapted for older adults (e.g. [26]). More recent developments of CBT are based on mindfulness, acceptance and values and might be particularly suitable when processing chronic illness, pain, loss or limited resources. Other recent approaches (e.g. cognitive behavioral analysis system of psychotherapy) are more eligible to improve interactional difficulties, as often present in the context of care.
*Problem solving therapy (PST)*	Variants of PST aim to reduce psychological distress. In a highly structured process, PST therapists develop patients’ problem solving capabilities by psychoeducation, interactive exercises, and motivational homework assignments. Thus, PST can support depressed home-living vulnerable patients in dealing with common stressors in their daily life (e.g. resulting from medical illness and limited resources) and meeting their needs. PST is also suitable for patients with cognitive impairments.
*Life review therapy (LRT)*	LRT is a form of reminiscence therapy in which patients are encouraged and systematically instructed to remember and articulate their memories in a structured and emotion-activating mode to deepen their self-knowledge, self-acceptance and integrity. Furthermore, they are supported in identifying biographical coping skills as well as developing a life balance and meaning.
*Interpersonal therapy (IPT)*	IPT is focused on solving interpersonal problems which often precede depression. It postulates four interpersonally relevant problem areas: role transitions, grief, interpersonal role dispute and interpersonal deficits. To improve communication skills and interpersonal functioning communication analyses, role play and coaching can be used. A wide range of interpersonal conflicts in the context of long-term home healthcare can be assigned to one of those problem areas (e.g. end of working life, loss of own home, conflict with caregivers) and thus be addressed with IPT.
*Psychodynamic psychotherapy (PDT)*	Variants of (short-term) PDT focus on the therapeutic relationship to gain insight into biographical causes and unconscious intrapsychic and intrapersonal conflicts that drive their symptoms or maladaptive functioning. General PDT techniques (e.g. exploratory inquiry, interpretation and clarification) can be used in older adults, with limitation to sufficient cognitive capacity of the client. More recent PDT developments take late life development goals into account. Supportive and containing strategies address older patients’ need for support in emotional regulation.

Further psychotherapeutic advances and modifications in depression treatment for specific vulnerable patient populations have been researched, including those with sensory limitations, specific chronic conditions and cognitive impairment. Procedural adaptations for sensory limitations may be required for some patients but should not automatically be assumed. For example, therapists should compensate actual hearing deficits by facing the patient, speaking clearly and emphasizing consonants but should be sensitive to avoid unnecessary accommodations of speech (secondary baby talk), based on age-associated stereotypes [21]. Psychotherapeutic interventions for patients with specific comorbid chronic conditions, such as diabetes, Parkinson’s disease, chronic obstructive pulmonary disease or heart failure, mostly aim to enhance self-efficacy and adherence to treatment or rehabilitation, self-care and functioning as well as patients’ acceptance of their illness and associated functional impairment (for an overview see [34]). For treating depression in older adults with mild cognitive impairment and early stages of dementia, empirically validated approaches based on standard psychotherapy have been developed, mostly utilizing PST and the involvement of caregivers to improve mental health, functional status and coping skills [33, 34]. Recommended procedural adaptations to address cognitive changes include multimodal presentation, repetition and summaries of significant information and the use of assistive devices (e.g. notebooks).

In patients with mild to moderate dementia cognitive stimulation therapy (CST) has been found to improve general well-being and quality of life. The CST is a group or individual intervention program that provides stimulating pleasurable activities and can be applied by certified professional caregivers [1, 45]. Although CST is not specifically aimed at the reduction of depressive symptoms, it might reduce emotional stress related to dementia by positive effects on social interaction and the quality of the relationship with caregivers [43].

As many vulnerable older adults suffer from more than one sensory, medical or cognitive condition as well as mobility limitations, programs addressing this undersupplied patient group should ideally cover a wide array of treatment demands simultaneously. In the following, two interventions that explicitly include care-dependent, cognitively and functionally impaired older adults are highlighted: PATH [24] as an international program catering to the needs of cognitively impaired vulnerable older people with a high degree of empirical evidence and replication, as well as PSY-CARE [15], an innovative German program, which is unique in the amount and high quality of psychotherapeutic intervention tailored to the needs of care-dependent vulnerable older adults.

The well-researched American PATH program [24] has been tailored to meet the needs of community-dwelling older adults with depression, cognitive impairment and limited mobility. The intervention aims to improve adaptive functioning and to alleviate depression. In the PATH program, home-delivered PST is supplemented with compensatory strategies, environmental adaptation tools and caregiver participation. Clinicians work with patients using an individualized, structured problem solving approach (e.g. plan and execute pleasurable activities, direct attention, change cognitions). Environmental adaptation tools (e.g. calendars, alarms, notes) as well as involvement of caregivers, support patients to implement plans and reduce environmental stress thus improving functioning and self-efficacy. In addition to increased accessibility for patients with limited mobility, the home delivery enables in situ exploration and implementation of compensatory strategies and environmental adaptations. In a randomized controlled trial (RCT) with 74 participants, PATH was compared to a nonspecific supportive therapy (e.g. empathic listening, establishing optimism [23]). Both interventions were well accepted but compared to those receiving supportive therapy, the PATH treatment yielded a greater decline in depression (43%) and disability (93%), statistically as well as clinically. This supports the efficacy of the program and collaborative care approaches for addressing the complex needs of this patient group.

The currently ongoing PSY-CARE trial [15], an innovative approach from Germany, has been tailored to specifically meet the needs of home-living vulnerable older adults with depression in need of care. The PSY-CARE trial aims to reduce depressive symptoms, improve overall quality of life and global functioning. The psychotherapeutic treatment conveyed by PSY-CARE is provided by specifically trained and supervised psychotherapists as a part of regular German healthcare. Psychotherapies are guided by a manual which uses CBT as a framework and is augmented by LRT techniques. Furthermore, the manual encourages psychotherapists to reflect on their own images of ageing by discussing the therapeutic attitude and defining guidelines. Home-delivered psychotherapy, collaboration with healthcare providers of different professions as well as involvement of caregivers is explicitly encouraged. The ongoing RCT compares the efficacy of the PSY-CARE treatment to a control group receiving brief psychosocial counselling. Participants aged 60 years and older (*N* = 197) with clinically significant depressive symptoms who were assigned a long-term care grade (assessed by the German statutory nursing insurance) have been recruited either via self-referral or gatekeeper referral. Most patients receive 12–24 sessions. Participants in the control group receive a self-help guide as well as individual telephone counselling, provided by trained psychologists.

## Psychotherapy in the context of collaborative care management

Interprofessional collaboration is a necessity when working with patients with complex health issues. Collaboration may be conducted via regular telephone conferences or shared databases. Alternatively, many programs utilize case managers (CM) as close and regular contact to the patient, deliverer of short psychotherapeutic interventions and link between professionals [6]. Some home healthcare companies in the USA employ their own mental health professionals as part of treatment teams (e.g. [11]) to increase access to mental health services. Studies support the superiority of collaborative care compared to treatment as usual regarding alleviation of depression [10].

Stepped care approaches involve an initial screening, followed by tailoring the treatment to the initial intervention choice and response, escalating in specialization, complexity and cost if remission is not achieved. A particularly noteworthy collaborative and stepped-care program from the USA is the IMPACT study [40]. In this RCT, depressed patients were assigned to a trained CM under supervision of a primary care physician and a psychiatrist. Treatment consisted of either psychopharmacological medication or PST delivered by the CM and was switched or combined in cases of nonresponse. Further interventions, such as additional psychotherapy or hospitalization, were implemented if case remission could not be achieved. The IMPACT intervention significantly reduced depressive symptoms compared to treatment as usual. A German adaptation, the GermanIMPACT has been conducted as a cluster RCT [41] and yielded significantly positive results concerning depression remission rates compared to treatment as usual [17]. While the participating physicians perceived the program as reducing their workload, the general participation of primary care physicians was low.

## Telehealth technology use for homebound older adults

An alternative cost-effective treatment approach is using telehealth services. By delivering psychotherapy either online or via telephone, these interventions can play a significant part in overcoming barriers for utilizing psychotherapeutic care for older physically impaired people. Especially for rural dwelling patients or those with limited mobility, telehealth may facilitate improved access to an adequate treatment. Healthcare professionals view telehealth as a practical and economical way of delivering healthcare to older adults but a significant proportion of older adults lack access to the necessary technology [19]. Moreover, a systematic literature overview [16] emphasized that certain aspects, such as living arrangements, level of education and functionality, comfort with using the internet and satisfaction as well as experience with psychotherapy need to be considered when evaluating telehealth as an appropriate treatment approach. Especially in a psychotherapeutic context where the formation of a therapeutic alliance plays a central role, studies suggested that while such an alliance may be successfully formed, especially patients with neuropsychological deficits, those highly susceptible to distractions or frail patients tended to perceive the therapeutic relationship as weaker [38] while some patient groups may completely oppose tele-delivered psychotherapy [14]. Findings regarding the positive effects of telehealth interventions are inconsistent. Furthermore, there is a lack of reliable studies evaluating factors regarding the implementation of telehealth, especially for vulnerable older adults.

## Psychosocial support for vulnerable older adults in the time of COVID-19

The COVID-19 pandemic globally impacts all age groups, yet older people with chronic health conditions (e.g. hypertension, heart problems and diabetes) are at higher risk of severe progression and mortality if they contract the disease [44]. Because physical distancing is deemed effective in containing the spread of COVID-19, national health authorities suggest that chronically ill older adults confine themselves to their homes which can result in isolation. Furthermore, many vulnerable older adults depend on in-person support of caregivers or nursing services, which have been partially or wholly suspended due to containment measures. Thus, they have been cut off from essential healthcare. In response to perceived threats, social isolation and loneliness as well as negative ageism, many vulnerable older adults are currently experiencing increased levels of anxiety, stress and helplessness [4]. Additionally, deficit-oriented views on ageing as well as paternalistic discourse (e.g. depicting older people as frail, helpless and in need of rescue) may aggravate the feelings of incapacitation and worthlessness in older people, thus negatively impacting their mental health [8].

Psychosocial interventions must be implemented to bridge social isolation, detect illness and reach older adults struggling with their mental health during the pandemic [39]. Innovative telehealth approaches as well as COVID-19 support hotlines for older people are rapidly being developed all over the world. Yet they still require evaluation and funding. For example, the American telephone call outreach program “Seniors Overcoming Social Isolation” is one of the first evaluated programs that targets this issue. Healthcare providers referred older care-dependent adults who might be at risk of social isolation to the program [32]. Medical and nursing students proceeded to call these adults, aiming to provide support and to train their own age-specific and context-specific communication skills. Such interventions cannot replace psychotherapeutic treatment; however, following the idea of a stepped care approach, psychosocial support calls may address subclinical symptoms and initiate psychotherapy when further treatment is needed.

Because telehealth approaches are subject to the same general restrictions and access barriers mentioned previously, COVID-19 may widen inequalities caused by digital exclusion; however, the COVID-19 pandemic can also act as a catalyst to accelerate digital inclusion efforts and initiate forthcoming psychotherapeutic care strategies that have the potential to address unmet treatment needs of home-living vulnerable older adults even after times of COVID-19.

## Conclusion

- Psychotherapy is an efficacious alternative or supplement to pharmacotherapy for vulnerable older adults with complex health problems, multimorbidity or polypharmacy.
- Even though practitioners’ qualification as well as funding arrangements for psychotherapy vary widely between national healthcare systems, the international literature indicates a highly prevalent need to improve access to psychotherapy meeting the demands of home-living vulnerable older adults.
- Practitioners who offer psychotherapy need a higher level of flexibility regarding the setting (tele-delivery, home visits) and individual necessities as well as specific training opportunities to deliver age-sensitive psychotherapy.
- Health insurance providers should support and fund stepped care programs as well as necessary psychotherapeutic adaptations such as home visits. Practitioners should work collaboratively and instate a case manager.
- Vulnerable patients should be actively screened for mental health issues and engaged to utilize healthcare services, especially in times of crises, such as the COVID-19 pandemic.

## References

[1] Aguirre E, Woods RT, Spector A, Orrell M (2013). Cognitive stimulation for dementia: a systematic review of the evidence of effectiveness from randomised controlled trials. Ageing Res Rev.

[2] American Psychological Association (2014). Guidelines for psychological practice with older adults. Am Psychol.

[3] Atiq R (2006). Common themes and issues in geriatric psychotherapy. Psychiatry.

[4] Banerjee D (2020). The impact of Covid-19 pandemic on elderly mental health. Int J Geriatr Psychiatry.

[5] Bharucha AJ, Dew MA, Miller MD, Borson S, Reynolds C (2006). Psychotherapy in long-term care: a review. J Am Med Dir Assoc.

[6] Bjerregaard F, Hüll M, Stieglitz RD, Hölzel LP (2018). Zeit für Veränderung – Was wir für die hausärztliche Versorgung älterer depressiver Menschen von den USA lernen können [Time for change: what we can learn from the USA about primary care of the depressed elderly. Gesundheitswesen.

[7] Blackburn P, Wilkins-Ho M, Wiese B (2017). Depression in older adults: diagnosis and management. BC Med J.

[8] Brooke JJ, Jackson D (2020). Older people and COVID-19: isolation, risk and ageism. J Clin Nurs.

[9] Carpenter B, Ruckdeschel K, Ruckdeschel H, Van Haitsma K, Norris MPVMSO-H (2002). R-E-M Psychotherapy: A manualized approach for long-term care residents with depression and dementia. Emerging trends in psychological practice in long-term care.

[10] Chang-Quan H, Bi-Rong D, Zhen-Chan L, Yuan Z, Yu-Sheng P, Qing-Xiu L (2009). Collaborative care interventions for depression in the elderly. J Investig Med.

[11] Choi N, Marti C, Bruce M, Hegel M (2013). Depression in homebound older adults: problem-solving therapy and personal and social resourcefulness. Behav Ther.

[12] Choi N, Sirey J, Bruce M (2013). Depression in homebound older adults: Recent advances in screening and psychosocial interventions. Curr Transl Geriatr Exp Gerontol Rep.

[13] DGPPN B KA, AkdÄ B, BApK D, DEGAM D, DGPs D, Guideline Group Unipolar Depression (2015) S3-Leitlinie/Nationale Versorgungsleitlinie Unipolare Depression – Langfassung, 2. Auflage: Version 5. [S3-Guideline/National Disease Management Guideline Unipolar Depression—Long Version. 2nd edition: Version 5. https://www.awmf.org/uploads/tx_szleitlinien/nvl-005l_S3_Unipolare_Depression_2017-05.pdf. Accessed 4 June 2020. 10.6101/AZQ/000364

[14] Frost R, Beattie A, Bhanu C, Walters K, Ben-Shlomo Y (2019). Management of depression and referral of older people to psychological therapies: a systematic review of qualitative studies. Br J Gen Pract.

[15] Gellert P, Beyer AK, Tegeler C, Vathke C, Kuhlmey A, Kessler EM (2020). Outpatient psychotherapy for home-living vulnerable older adults with depression: study protocol of the PSY-CARE trial. BMC Geriatr.

[16] Harerimana B, Forchuk C, O’Regan T (2019). The use of technology for mental healthcare delivery among older adults with depressive symptoms: a systematic literature review. Int J Mental Health Nurs.

[17] Hölzel LP, Bjerregaard F, Bleich C, Boczor S, Härter M, König HH, Kloppe T, Niebling W, Scherer M, Tinsel I, Hüll M (2018). Coordinated treatment of depression in elderly people in primary care. Dtsch Arztebl Int.

[18] Huang AX, Delucchi K, Dunn LB, Nelson JC (2015). A systematic review and meta-analysis of psychotherapy for late-life depression. Am J Geriatr Psychiatry.

[19] Hunsaker A, Hargittai E (2018). A review of internet use among older adults. N Media Soc.

[20] Kessler EM, Blachetta C (2020). Age cues in patients’ descriptions influence treatment attitudes. Aging Ment Health.

[21] Kessler EM, Bowen CE (2015). Images of aging in the psychotherapeutic context. GeroPsych.

[22] Kessler EM, Tegeler C (2018). Psychotherapeutisches Arbeiten mit alten und sehr alten Menschen [Psychotherapeutic work with old and very old people. Psychotherapeut.

[23] Kiosses DN, Ravdin LD, Gross JJ, Raue P, Kotbi N, Alexopoulos GS (2015). Problem adaptation therapy for older adults with major depression and cognitive impairment: a randomized clinical trial. JAMA Psychiatry.

[24] Kirkham J, Seitz D, Choi N (2015). Meta-analysis of problem solving therapy for the treatment of depression in older adults. Am J Geriatr Psychiatry.

[25] Koder DA, Helmes E (2006). Clinical psychologists in aged care in Australia: a question of attitude or training?. Aust Psychol.

[26] Konnert C, Petrovic-Poljak A (2014). Barriers to mental healthcare utilization. The Oxford handbook of clinical geropsychology. Oxford library of psychology.

[27] Laidlaw K, Kishita N (2015). Age-appropriate augmented cognitive behavior therapy to enhance treatment outcome for late-life depression and anxiety disorders. GeroPsych.

[28] Luck-Sikorski C, Stein J, Heilmann K, Maier W, Kaduszkiewicz H, Scherer M, Weyerer S, Werle J, Wiese B, Moor L, Bock JO, König HH, Riedel-Heller SG (2017). Treatment preferences for depression in the elderly. Int Psychogeriatr.

[29] Luppa M, Sikorski C, Luck T, Ehreke L, Konnopka A, Wiese B, Weyerer S, König HH, Riedel-Heller SG (2012). Age- and gender-specific prevalence of depression in latest-life. Systematic review and meta-analysis. J Affect Disord.

[30] McGovern AR, Kiosses DN, Raue PJ, Wilkins VM, Alexopoulos GS (2014). Psychotherapies for late-life depression. Psychiatr Ann.

[31] Meeks S, Looney SW, Van Haitsma K, Teri L (2008). BE-ACTIV: A staff-assisted behavioral intervention for depression in nursing homes. Geront.

[32] Office EE, Rodenstein MS, Merchant TS, Pendergrast TR, Lindquist LA (2020). Reducing social isolation of seniors during COVID-19 through medical student telephone contact. J Am Med Dir Assoc.

[33] Orgeta V, Qazi A, Spector AE, Orrell M (2014). Psychological treatments for depression and anxiety in dementia and mild cognitive impairment. Cochrane Database Syst Rev.

[34] Raue PJ, McGovern AR, Kiosses DN, Sirey JA (2017). Advances in psychotherapy for depressed older adults. Curr Psychiatry Rep.

[35] Read JR, Sharpe L, Modini M, Dear BF (2017). Multimorbidity and depression: a systematic review and meta-analysis. J Affect Disord.

[36] Soysal P, Veronese N, Thompson T, Kahl KG, Fernandes BS, Prina AM, Solmi M, Schofield P, Koyanagi A, Tseng PT, Lin PY, Chu CS, Cosco TD, Cesari M, Carvalho AF, Stubbs B (2017). Relationship between depression and frailty in older adults: a systematic review and meta-analysis. Ageing Res Rev.

[37] Tedeschini E, Levkovitz Y, Iovieno N, Ameral VE, Nelson JC, Papakostas GI (2011). Efficacy of antidepressants for late-life depression: a meta-analysis and meta-regression of placebo-controlled randomized trials. J Clin Psychiatry.

[38] Titov N, Dear BF, Ali S, Zou JB, Lorian CN, Johnston L, Terides MD, Kayrouz R, Klein B, Gandy M, Fogliati VJ (2015). Clinical and cost-effectiveness of therapist-guided internet-delivered cognitive behavior therapy for older adults with symptoms of depression: a randomized controlled trial. Behav Ther.

[39] Humanitarian UN (2020) COVID-19: Older people at risk but resilient. https://unocha.exposure.co/covid19-older-people-at-risk-but-resilient. Accessed 19 June 2020

[40] Unützer J, Katon W, Callahan CM, Williams J, John W, Hunkeler E, Harpole L, Hoffing M, Penna DRD, Noël PH, Lin EHB, Areán PA, Hegel MT, Tang L, Belin TR, Oishi S, Langston C, IMPACT Investigators (2002). Collaborative care management of late-life depression in the primary care setting: a randomized controlled trial. JAMA.

[41] Wernher I, Bjerregaard F, Tinsel I, Bleich C, Boczor S, Kloppe T, Scherer M, Härter M, Niebling W, König HH, Hüll M (2014). Collaborative treatment of late-life depression in primary care (GermanIMPACT): study protocol of a cluster-randomized controlled trial. Trials.

[42] Wilson KC, Mottram PG, Vassilas CA (2008). Psychotherapeutic treatments for older depressed people. Cochrane Database Syst Rev.

[43] Woods B, Aguirre E, Spector AE, Orrell M (2012). Cognitive stimulation to improve cognitive functioning in people with dementia. Cochrane Database Syst Rev.

[44] World Health Organization (2020) Health care considerations for older people during COVID-19 pandemic. https://www.euro.who.int/en/health-topics/health-emergencies/coronavirus-covid-19/technical-guidance/health-care-considerations-for-older-people-during-covid-19-pandemic. Accessed 24 June 2020

[45] Yates LA, Leung P, Orgeta V, Spector A, Orrell M (2015). The development of individual cognitive stimulation therapy (iCST) for dementia. Clin Interv Aging.

[46] Zivin K, Wharton T, Rostant O (2013). The economic, public health, and caregiver burden of late-life depression. Psychiatr. Clin. North Am..

